# MiR‐205 promotes endothelial progenitor cell angiogenesis and deep vein thrombosis recanalization and resolution by targeting PTEN to regulate Akt/autophagy pathway and MMP2 expression

**DOI:** 10.1111/jcmm.14739

**Published:** 2019-10-21

**Authors:** Li‐Li Sun, Lun Xiao, Xiao‐Long Du, Lei Hong, Cheng‐Long Li, Jian Jiao, Wen‐Dong Li, Xiao‐Qiang Li

**Affiliations:** ^1^ Department of Vascular Surgery The Affiliated Drum Tower Hospital Nanjing University Medical School Nanjing China; ^2^ Department of Vascular Surgery The Second Affiliated Hospital of Soochow University Suzhou China; ^3^ Department of Vascular Surgery Fengyang County People's Hospital Chuzhou China

**Keywords:** angiogenesis, deep vein thrombosis, endothelial progenitor cell, microRNA, migration

## Abstract

MicroRNAs (MiRNAs, MiRs) represent a class of conserved small non‐coding RNAs that affect post‐transcriptional gene regulation and play a vital role in angiogenesis, proliferation, apoptosis, migration and invasion. They are essential for a wide range of physiological and pathological processes, especially for vascular diseases. However, data concerning miRNAs in endothelial progenitor cells (EPCs) and deep vein thrombosis (DVT) remain incomplete. We explored miRNAs that modulate angiogenesis in EPCs and thrombolysis, and analysed their underlying mechanisms using a DVT model, dual‐luciferase reporter assay, qRT‐PCR, Western blot, immunofluorescence staining, flow cytometry analysis, CCK‐8 assay, angiogenesis assay, wound healing and Transwell assay. We found that miR‐205 enhanced the homing ability of EPCs to DVT sites and promoted thrombosis resolution and recanalization, which significantly reduced venous thrombus. Additionally, we demonstrated that miR‐205 overexpression significantly enhanced angiogenesis in vivo and in vitro, migration, invasion, F‐actin filaments and proliferation in EPCs, and inhibited cell apoptosis. Conversely, down‐regulation of miR‐205 played the opposite role in EPCs. Importantly, this study demonstrated that miR‐205 directly targeted PTEN to modulate the Akt/autophagy pathway and MMP2 expression, subsequently playing a key role in EPC function and DVT recanalization and resolution. These results elucidated the pro‐angiogenesis effects of miR‐205 in EPCs and established it as a potential target for DVT treatment.

## 1 INTRODUCTION

Deep vein thrombosis (DVT) refers to the formation of blood clots in deep venous lumen, causing blood flow disorders, and its prevalence increases with age.^1^, ^2^ DVT with its major complications, post‐thrombotic syndrome (PTS) and pulmonary embolism (PE) can be life‐threatening, which is a global health problem.^3^, ^4^, ^5^ PTS consists of pain, swelling and even ulceration of the leg that can occur immediately after DVT or can delay the onset.^6^, ^7^, ^8^ Prophylaxis and treatments for DVT patients include anticoagulation by heparin, vitamin K antagonists, and thrombin and Factor Xa inhibitors. Deep vein thrombosis surgical intervention consists of thrombectomy or catheter‐based thrombolysis using urokinase, streptokinase or tissue plasminogen activator.^9^ Unfortunately, these methods are not always effective. Studies have shown that the main consequences of DVT are death, recurrence, PTS and major bleeding caused by anticoagulation.^4^, ^10^ Thus, it is essential to study the molecular mechanism of DVT and explore alternative therapies for DVT treatment.

Of note, angiogenesis and thrombolysis are crucial for thrombus recanalization.^11^, ^12^, ^13^ Numerous studies have shown that endothelial progenitor cells (EPCs) are recruited to the thrombus to accelerate thrombus resolution and play a vital role in physiological and pathological neovascularization in adults.^12^, ^14^, ^15^, ^16^ Moreover, EPCs can differentiate into endothelial cells to participate in angiogenesis and thus are a promising therapeutic approach for thrombus resolution as there is only limited success with the current treatment options.^12^, ^16^, ^17^ However, the function of EPCs can be impaired by cardiovascular risk factors, ischaemic disease and graft vasculopathy.^18^, ^19^, ^20^, ^21^ Therefore, it is important to explore methods to improve EPC homing and angiogenesis.

MicroRNAs (MiRNAs, MiRs) are small non‐coding RNAs consisting of approximately 22 nucleotides that influence post‐transcriptional gene regulation by targeting the 3′ untranslated regions (3′UTRs) of mRNAs.^22^, ^23^, ^24^, ^25^ They play a major role in the regulation of cell migration, proliferation, apoptosis and angiogenesis, which is essential for the development and progression of vascular disease.^26^ Our previous studies have suggested that miRNAs including miR‐483‐3p, miR‐150, let‐7e‐5p and miR‐126 play a crucial role in regulating angiogenesis and migration in EPCs.^27^, ^28^, ^29^, ^30^ A recent study has also indicated that miR‐126‐loaded EPC‐derived exosomes are a promising potential vehicle for DVT therapy.^11^ Among the emerging miRNAs, miR‐205 has an important impact on the progression of diseases, but it is a “double‐edged sword” in diseases. Studies have shown that miR‐205 can facilitate wound healing in human corneal epithelial cells,^31^ while it promotes tumour angiogenesis and cancer cell proliferation and migration in human nasopharyngeal carcinoma, lung cancer, breast cancer and endometrial cancer.^32^, ^33^, ^34^, ^35^ On the contrary, miR‐205 has been shown to play an antiproliferation role in certain tumours.^36^ MiRNAs can cooperatively target a variety of mRNAs, and the same mRNA can be regulated by multiple miRNAs, and therefore, the role of miRNA may vary with cellular content and stimulation, which provides a possible explanation for the complex and even controversial functions of miR‐205. Recently, it has also been confirmed that miR‑205 promotes ovarian cancer cell invasion by enhancing the expression of matrix metalloproteinase (MMP) 2/9.^37^ MMP2 is an essential factor for promoting EPC migration and early venous thrombus resolution.^38^, ^39^, ^40^ However, the role and underlying mechanism of miR‐205 in DVT and EPCs, particularly in regulating angiogenesis and thrombus resolution, remain largely unknown.

Here, we showed the role of miR‐205 in EPCs and DVT recanalization and resolution, and explored the underlying molecular mechanism, which is crucial in the pathogenesis of DVT and contributes to the development of a novel effective treatment for DVT.

## 2 MATERIALS AND METHODS

### 2.1 Cell culture and transfection

Endothelial progenitor cells were cultured in endothelial basal medium‐2 (EBM‐2; Lonza) and EGM‐2 MV SingleQuot Kit Suppl. & Growth Factors (Lonza) and maintained at 37°C in a 5% CO_2_ incubator. Lentiviral particles carrying vehicle control (NC), miR‐205 mimics and inhibitor were provided by GenePharma. They were used to infect EPCs according to the manufacturer's instructions.

### 2.2 Quantitative real‐time PCR analysis

Total RNA was extracted by TRIzol reagent (Invitrogen). The PrimeScript RT reagent kit (TaKaRa) was used for first strand of cDNA synthesis, and the SYBR Green quantitative PCR Master Mix (Bio‐Rad) was used for gene amplification. MiRNA levels were determined following the instructions of the Hairpin‐it™ miRNAs RT‐PCR Quantitation Kit (GenePharma). MRNA expression levels were analysed using mRNA primers (GENEWIZ). The endogenous control for miR‐205 was *U6* RNA, and for the other transcripts, it was *GAPDH*. Quantitative real‐time PCR (qRT‐PCR) was performed on a High‐Throughput Quantitative PCR LightCycler480 II (Roche Applied Science).

### 2.3 In vivo angiogenesis assay

Male nude mice (5 weeks old) were provided by Shanghai Lingchang Biotechnology Co., Ltd. and raised in the Experimental Animal Center of Soochow University. Endothelial progenitor cells (5 × 10^5^) were mixed with 200 μL of Matrigel (BD) and injected subcutaneously into nude mice. Matrigel implants were removed and used to analyse angiogenesis by haematoxylin and eosin (H&E) staining. Images were obtained by an Olympus DP73 microscope.

### 2.4 In vitro angiogenesis assay

Matrigel (50 μL/well) was transferred to a 96‐well plate, followed by inoculation of transfected EPCs (3 × 10^4^ cells). After 12 hours, images were captured with an inverted microscope. The extent of tube formation was assessed by measuring the tube number using Image J software.

### 2.5 Analysis of the therapeutic effect on deep vein thrombosis

Male nude mice (10 weeks old) were anesthetized, and the left jugular vein was isolated under a stereo microscope, and the proximal end of the heart and its branches were ligated. GFP‐miR‐205 EPCs and GFP‐NC EPCs (5 × 10^6^) were injected into the nude mice through the orbital vein at 48 hours after DVT. After 7 days, thrombi with vessel walls were harvested for measurements of size and weight, and for immunofluorescence. The number of GFP‐EPCs at the thrombus was observed under an IX‐81 laser confocal microscope (Olympus) to analyse homing ability.

### 2.6 Migration assay

Endothelial progenitor cells were seeded in 6‐well plates (3 × 10^5^ cells/well), and a 200‐µL pipette tip was used to create a linear scratch at nearly 95% confluence. Subsequently, fresh EBM‐2 medium without FBS was added to prevent EPC proliferation. Images were captured by an inverted microscope at 0 and 12 hours, and the number of migration cells was assessed by Image J and GraphPad Prism software.

### 2.7 Invasion assay

Corning Transwell chamber in 24‐well plates was used to perform cell invasion assays. Matrigel was added to the upper chamber and placed at 37°C for 30 minutes, followed by the addition of cells. EBM‐2 medium supplemented with 10% FBS was transferred to the lower compartment of the chamber. Images of invasion cells were obtained using an inverted microscope. The invasion assay analysis was performed using Image J software and GraphPad Prism software.

### 2.8 Cell proliferation and apoptosis assays

Cell Counting Kit‐8 (CCK‐8, Dojindo) was used for cell proliferation assay according to the specification. Briefly, transfected EPCs were seeded in 96‐well plates (3 × 10^3^ cells/well) and examined at 0, 24, 48, 72 and 96 hours. The detection buffer (100 μL, ratio of medium to CCK‐8 was 9:1) was added to each well at different time‐points. After incubation for 2 hours at 37°C, the absorbance at 450 nm was detected by Multi‐Detection Readera (SpectraMax5). For cell apoptosis analysis, the APC Annexin V Apoptosis Detection Kit with 7‐AAD (BioLegend) was used according to the instructions. All the tests were performed at least three times.

### 2.9 Immunofluorescence analysis

Endothelial progenitor cells were fixed with 4% paraformaldehyde and blocked with Immunol Staining Blocking Buffer (Beyotime, P0102). Immunostaining was performed using TRITC Phalloidin (Solarbio, CA1610, 1:2000) to identify F‐actin. For immunostaining of CD34 and MMP2, frozen sections of thrombus were stained with anti‐CD34 (Abcam, 1:100) or anti‐MMP2 (Abcam, 1:200). After incubation with fluorescent‐labelled secondary antibodies (donkey anti‐rabbit Alexa Fluor® 647, Abcam, 1:500), images were acquired using a confocal microscope under the same conditions for each experiment, and no digital manipulation of images was performed.

### 2.10 Luciferase activity assay

For the luciferase reporter assay, cells were cotransfected with firefly luciferase reporter plasmid (PTEN‐WT, PTEN‐WUT) and a Renilla luciferase vector (pMIR‐Report Luciferase, Promega) plus negative control and small RNAs (NC, miR‐205 mimics) using HiTrans™ LipoPlus Reagent (SYNTHGENE). Firefly and Renilla luciferase activities were analysed with Multi‐Detection Readera (SpectraMax5). Experiments were performed at least three times.

### 2.11 Western blot analysis

Total cellular protein was lysed in RIPA buffer supplemented with protease inhibitors, phosphatase inhibitors and PMSF, and centrifuged at 12 000 *g* for 10 minutes at 4°C. The BCA Protein Assay Kit (Beyotime, P0011) was used to quantify protein levels. Cellular proteins were separated by 10% or 15% SDS‐PAGE gels and transferred to polyvinylidene difluoride membranes (Bio‐Rad). The membranes were incubated with antibodies against PTEN [Cell Signaling Technology (CST), 1:1000], p‐AKT (CST, 1:1000), AKT (CST, 1:1000), MMP2 (Abcam, 1:1000), p62 (CST, 1:1000), LC3 I/II (CST, 1:1000) and β‐actin (Beyotime, 1:1000). IRDye® 800CW goat anti‐rabbit or antimouse IgG antibodies (LI‐COR) were used at 1:10 000 dilution. The immune bonds were detected using an Infrared Imaging System (LI‐COR).

### 2.12 Statistical analysis

All data are presented as mean ± SEM. Student's *t* test and one‐way ANOVA were used to analyse significant differences between groups. GraphPad Prism software was used for statistical analysis. *P* < .05 was considered statistically significant.

## 3 RESULTS

### 3.1 MiR‐205 promotes EPC homing to the thrombus, and deep vein thrombosis recanalization and resolution

**Figure 1:**
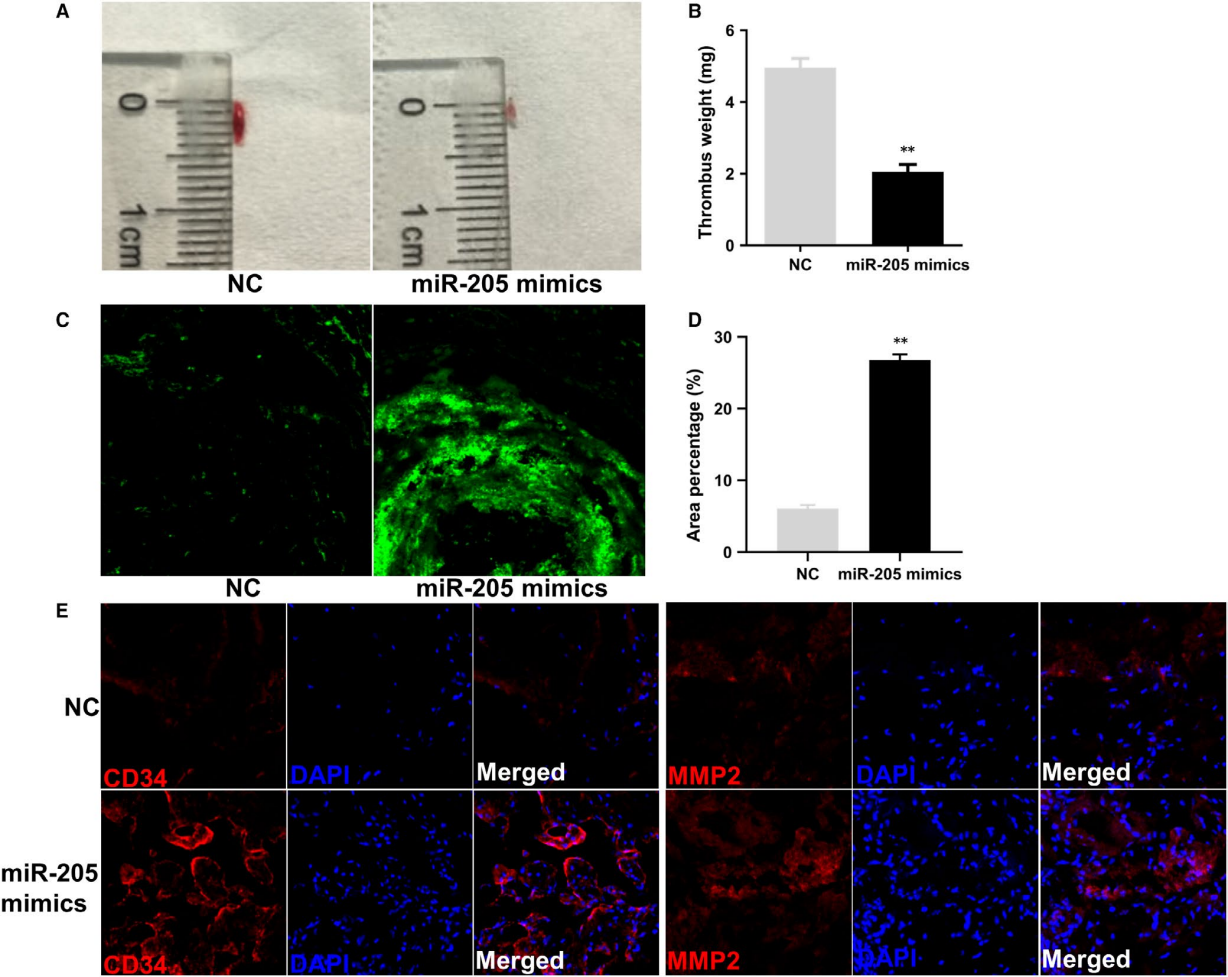
MiR‐205 reduces DVT size and weight, and promotes EPC homing and DVT recanalization and resolution. A‐B, Representative thrombus size and weight 7 d after the injection of EPCs are shown. Relative thrombus weight change is expressed as mean ± SEM. **P < .01. C‐D, The ability of EPC homing to the thrombus 7 d after injection of EPCs. Representative results are shown (magnification ×400). Area percentage of fluorescence represents the number of homing EPCs. **P < .01. E, Expression of CD34 (red) and MMP2 (red) in thrombus. Blue represents DAPI. Representative images are shown (magnification ×400)

Endothelial progenitor cells exert an important role in DVT mainly by regulating angiogenesis and thrombolysis.^13^ However, the function of EPCs can be impaired by a poor microenvironment in the body. In this study, a model of DVT was established by ligation of a jugular vein and its branches. Endothelial progenitor cells infected with NC or miR‐205 mimics were intravenously administrated into nude mice. The results showed that the thrombus size and weight were significantly reduced on day 7 after injection of GFP‐miR‐205 EPCs compared with GFP‐NC EPCs (*P* < .01, Figure 1A,B). Furthermore, EPCs homing to the thrombus significantly increased in the nude mice injected with GFP‐miR‐205 EPCs compared with GFP‐NC EPCs (*P* < .01, Figure 1C,D). In addition, immunostaining of venous thrombus (with the vein wall) showed nucleated cells, including mainly the injected EPCs, inflammatory cells and endothelial cells accumulating in the thrombus, MMP2 and CD34 expression levels. There were more nucleated cells, and the expression of CD34 and MMP2 was up‐regulated in nude mice injected with GFP‐miR‐205 EPCs, compared with the NC group (Figure 1E). Consistently, the up‐regulation of CD34 and MMP2 in thrombosis was in good agreement with the results showing promotion of recanalization and venous thrombus resolution. Overall, the results demonstrated that miR‐205 inhibited the thrombus size and weight, and promoted EPC homing to the thrombus, and DVT recanalization and resolution.

### 3.2 MiR‐205 promotes EPC angiogenesis in vivo and in vitro

**Figure 2:**
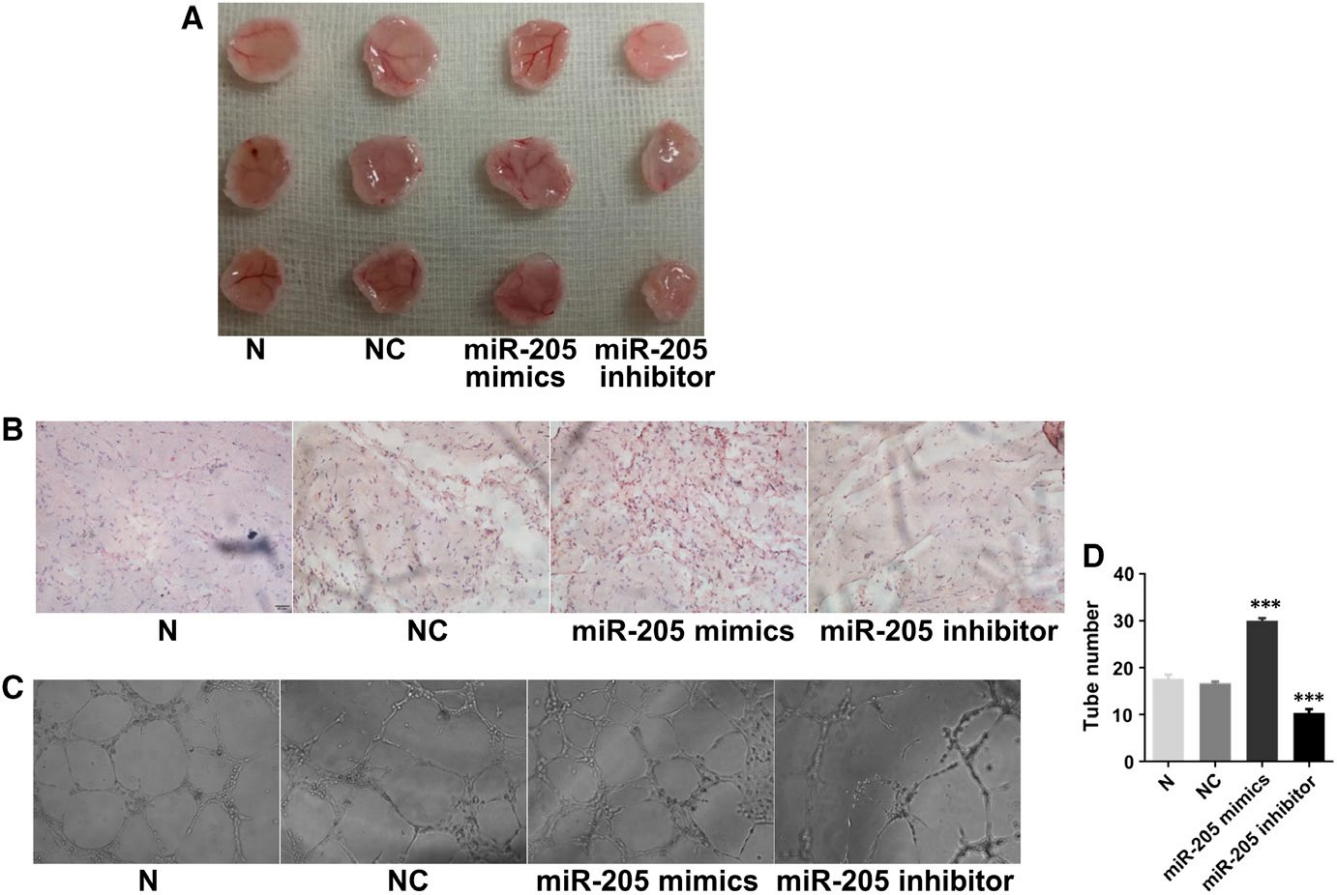
Effects of miR‐205 on angiogenesis in vivo and in vitro. A, In vivo angiogenesis was evaluated at Day 7 after subcutaneous injection of Matrigel‐mixed EPCs into nude mice. B, Haematoxylin and eosin (H&E) staining: miR‐205 overexpression and knockdown, respectively, increased and decreased tube formation by EPCs in vivo (magnification, ×200). C, Effects of miR‐205 on tube formation in EPCs (magnification, ×100). D, MiR‐205 overexpression and knockdown, respectively, increased and decreased tube number. ***P < .001 compared with NC group

The role of EPCs in DVT recanalization and resolution is associated with their migration, invasion and angiogenesis abilities. In the miR‐205 overexpression group, angiogenesis in the thrombus was enhanced. To determine whether miR‐205 functions in EPCs, we examined angiogenesis in vivo and in vitro of EPCs that were performed gene overexpression and knockdown via lentiviral infection. The result of angiogenesis in vivo showed that the implants with EPCs in miR‐205 mimics groups were redder in overall appearance than the NC groups, while the implants with EPCs in miR‐205 inhibitor groups were less red compared with those of the NC groups, with no differences between the N group and NC group (*P* > .05, Figure 2A). Meanwhile, H&E staining showed more luminal structures in implants with miR‐205 mimics EPCs than in NC group and fewer luminal structures in implants with miR‐205 inhibitor EPCs than in NC group (Figure 2B). To confirm our results of angiogenesis in vivo, we next detected the effect of miR‐205 on tube formation of EPCs in vitro (Figure 2C). The results suggested that the tube formation of EPCs in miR‐205 mimics group was significantly increased, while angiogenesis in EPCs of miR‐205 inhibitor group was attenuated, and there were no differences between the N group and NC group (*P* > .05, Figure 2D). These results suggested that miR‐205 played a vital role in EPC‐associated angiogenesis.

### 3.3 MiR‐205 enhances EPC migration and invasion by regulating F‐actin and MMP2

**Figure 3:**
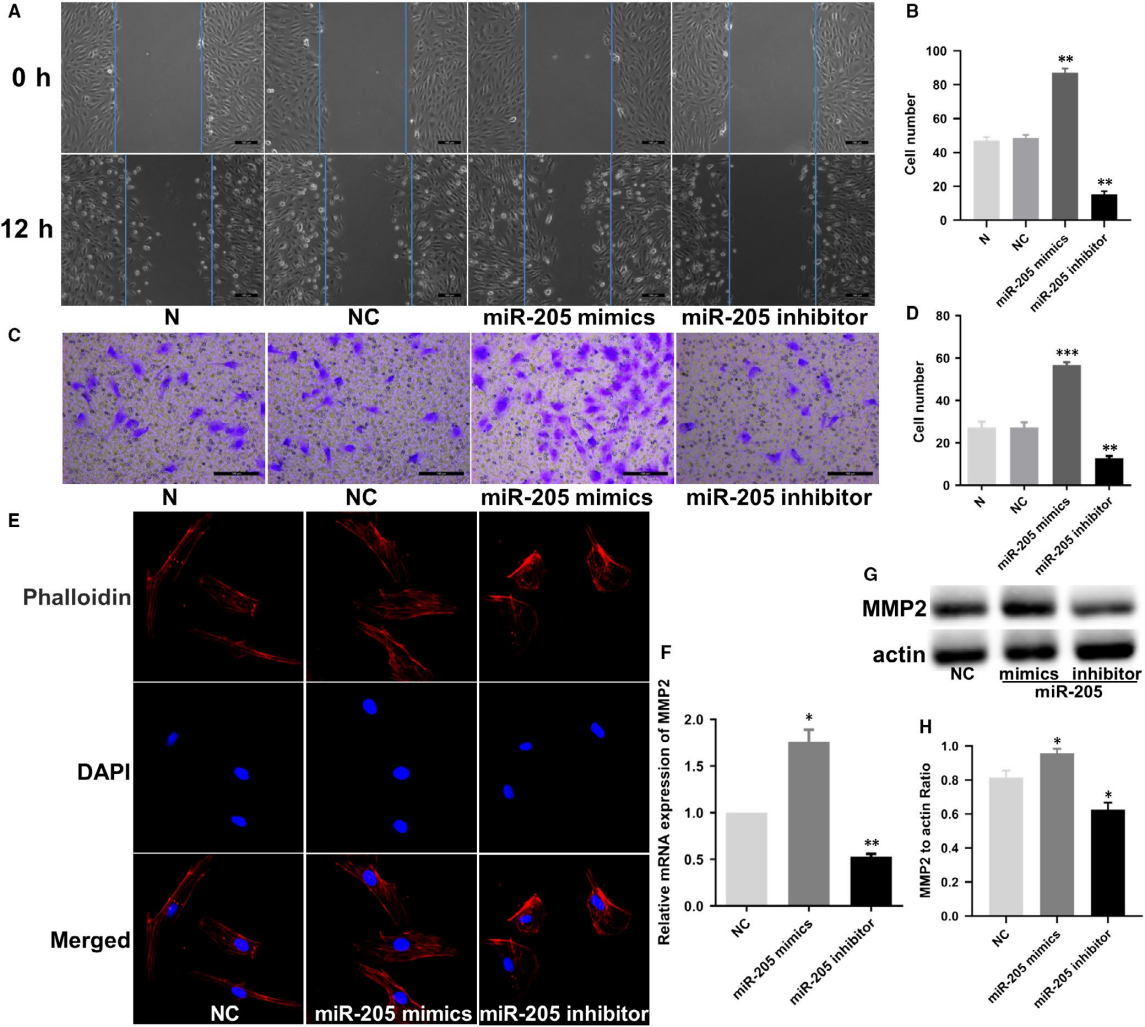
Effects of miR‐205 on cell migration and invasion in EPCs. A, Wound healing assay showing the effects of miR‐205 on EPC migration. Scale bar = 100 μm. MiR‐205 overexpression and knockdown, respectively, decreased and increased the cell number of EPC migrated. B, Effects of miR‐205 on cell migration ability were analysed. **P < .01 compared to the NC group. C, Transwell cell invasion assay provided results similar to those for wound healing. Scale bar = 100 μm. D, Effects of miR‐205 on cell invasion ability were analysed. **P < .01, ***P < .001 compared with NC group. E, Effects of miR‐205 on F‐actin in cultured EPCs. Cells were fixed, permeabilized, stained with rhodamine‐phalloidin and DAPI, and visualized by confocal microscopy; down‐regulation of miR‐205 impaired F‐actin filaments, while miR‐205 overexpression prevented disruption of F‐actin filaments. F‐H, MMP2 mRNA and protein expression levels were observed by qRT‐PCR and Western blot analysis. *P < .05, **P < .01 compared with NC group

To investigate effect of miR‐205 on EPC migration and invasion, wound healing and Transwell invasion assays were performed. Wound healing assay revealed that the ability of EPC migration in the miR‐205 mimics group was much higher than that in the NC group (*P* < .01), and the EPC migration ability of the miR‐205 inhibitor group was significantly reduced than that of the NC group (*P* < .01, Figure 3A,B). However, no significant difference was observed between untransfected and negative control cells (*P* > .05). Analogously, invasion assay shared similar results, whereby miR‐205 mimics increased cell invasion ability significantly (*P* < .001), while miR‐205 inhibitor obviously impaired the ability of cell invasion (*P* < .01), with no significant difference between N group and NC group (*P* > .05, Figure 3C,D). Further, F‐actin and MMP2, which are closely related to cell migration ability, were observed. As shown in Figure 3E, miR‐205 inhibitor impaired F‐actin filaments, while miR‐205 mimics prevented disruption of F‐actin filaments. Meanwhile, we found that the mRNA and protein expression levels of MMP2 increased in miR‐205 overexpression EPCs, which is consistent with the result of MMP2 expression in thrombus sites in mice injected with GFP‐miR‐205 EPCs, while the opposite results were observed in miR‐205 inhibitor group (Figure 3F‐H). These results disclosed that miR‐205 played a key regulatory role in EPC migration and invasion, at least in part, by regulating F‐actin filaments and MMP2 expression.

### 3.4 MiR‐205 regulates cell proliferation and apoptosis of EPCs

**Figure 4:**
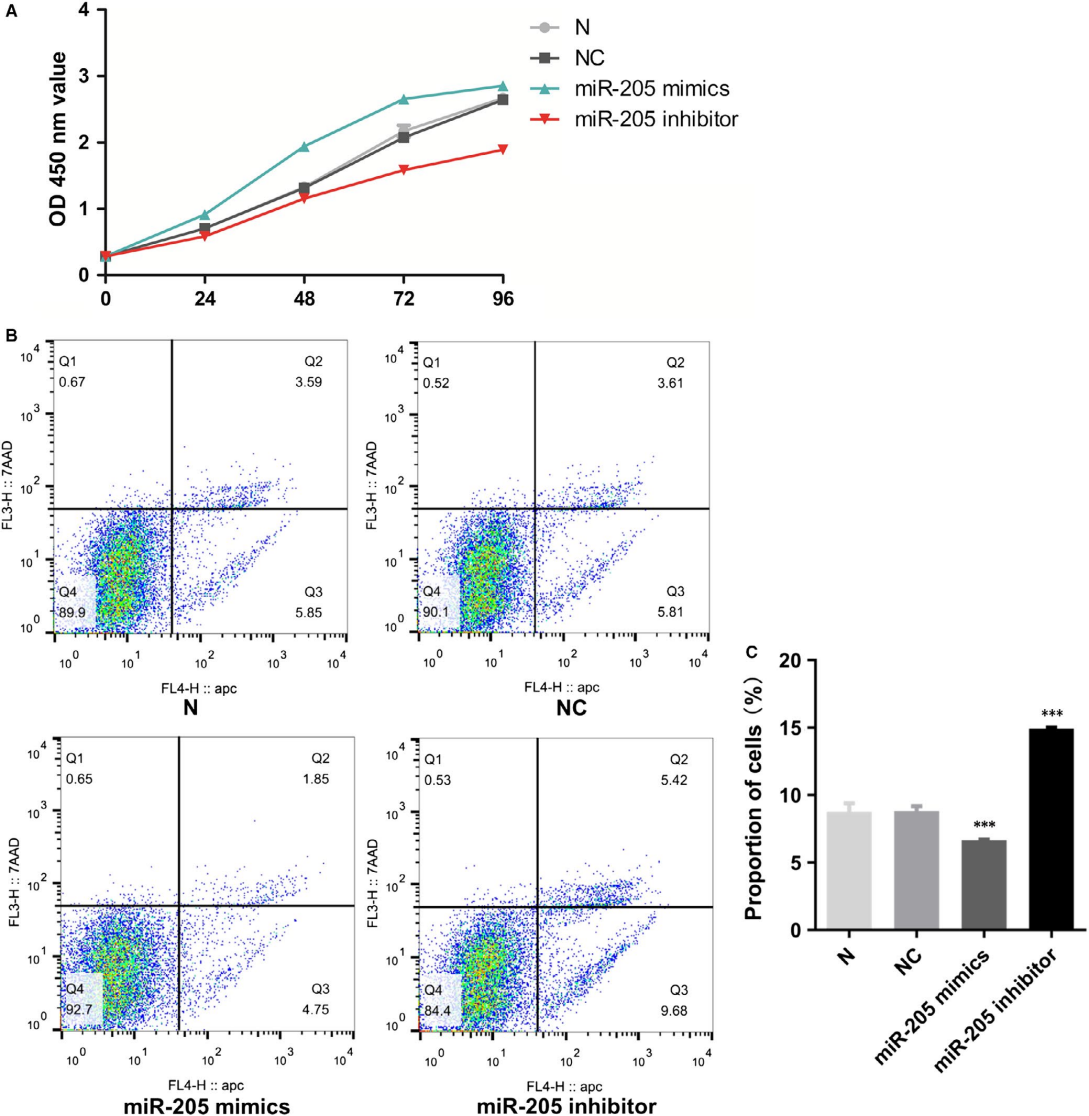
Role of miR‐205 in EPC growth. A, Cell proliferation ability of EPCs was detected by CCK8 assay. B‐C, Effects of miR‐205 on cell apoptosis by flow cytometry in three independent experiments. Data were presented as mean ± SD, ***P < .001

To test the role of miR‐205 in cell proliferation and apoptosis of EPCs, CCK‐8 assay and flow cytometry were performed. MiR‐205 mimics and inhibitor enhanced and suppressed, respectively, EPC proliferation compared with the NC group (Figure 4A). Cell apoptosis analysis revealed that the apoptotic cell population in miR‐205 overexpression EPCs was decreased, while the percentage of apoptotic cells in the miR‐205 inhibitor group was clearly increased compared with the NC group (Figure 4B,C). There was no significant difference in cell proliferation and apoptosis of EPCs between N group and NC group (*P* > .05). These data showed that miR‐205 functioned both as a cell growth inducer and as an apoptosis inhibitor in EPCs.

### 3.5 PTEN directly binds to miR‐205

**Figure 5:**
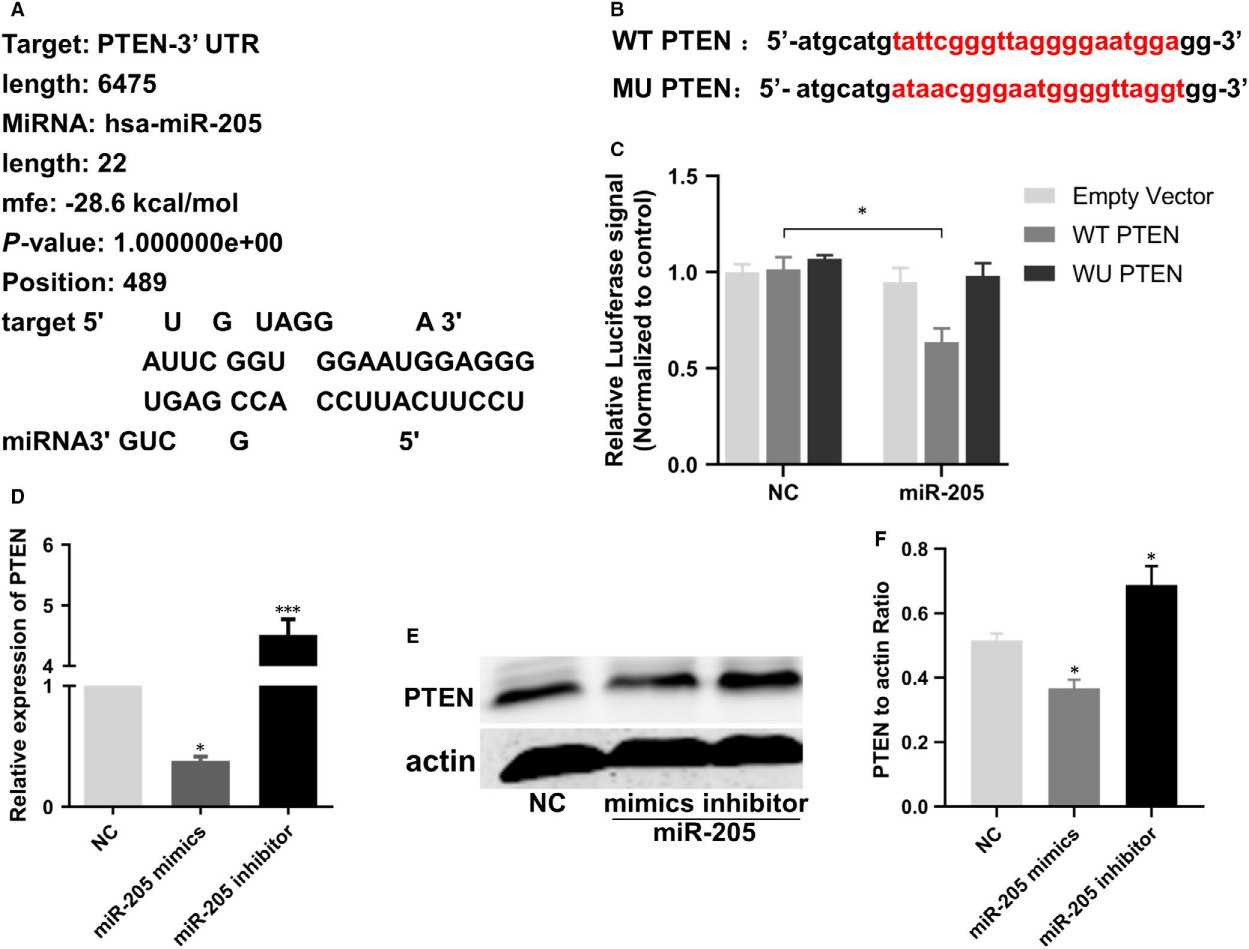
MiR‐205 directly bound with PTEN. A, Bioinformatics analysis revealed miR‐205 contains binding sequences complementary to the binding sites of PTEN. B, The luciferase reporter constructs containing WT‐PTEN and MU‐PTEN sequence. C, Dual‐luciferase reporter assay verified the targeting relationship between miR‐205 and PTEN. D, Relative quantification of mRNA levels of PTEN. *P < .05, ***P < .001. E‐F, Western blot revealed that miR‐205 overexpression and knockdown, respectively, decreased and increased protein expression of PTEN in EPCs. *P < .05 compared with NC group

Based on the miRNA database (http://www.mirbase.org), targetScan, RNA hybrid software and using bioinformatics methods to predict miR‐205 targets and analyse its functions, PTEN was found to be associated with miR‐205 and cell growth. Importantly, we demonstrated that the sequence of miR‐205 is complementary to the target sequence in the 3′UTR of PTEN and that their hybridization was predicted by RNA hybrid software, requiring a minimum free energy of –28.6 kcal/mol (Figure 5A). To further explore whether miR‐205 binds to the PTEN 3′UTR, the luciferase reporter assay was performed. As shown in Figure 5B, wild‐type (WT) PTEN and mutant (MU) PTEN were inserted into a luciferase reporter plasmid cleaved with HindIII and SpeI (TaKaRa) endonucleases. The dual‐luciferase reporter assay showed that only the group cotransfected with the WT PTEN plasmid and the miR‐205 mimics had decreased luciferase activity, while the other groups did not change significantly (Figure 5C), confirming that miR‐205 interacts with the 3′UTR of PTEN and that the binding region is consistent with the prediction. Furthermore, qRT‐PCR and Western blot analyses suggested that the PTEN mRNA and protein expression levels were reduced in the miR‐205 mimics group, whereas they were increased in the miR‐205 inhibitor group, compared with the NC group (Figure 5D‐F). Taken together, we concluded that PTEN was a direct target of miR‐205 in the regulation of EPCs.

### 3.6 Effects of cotransfection of miR‐205 and PTEN on tube formation, cell migration and invasion of EPCs

**Figure 6:**
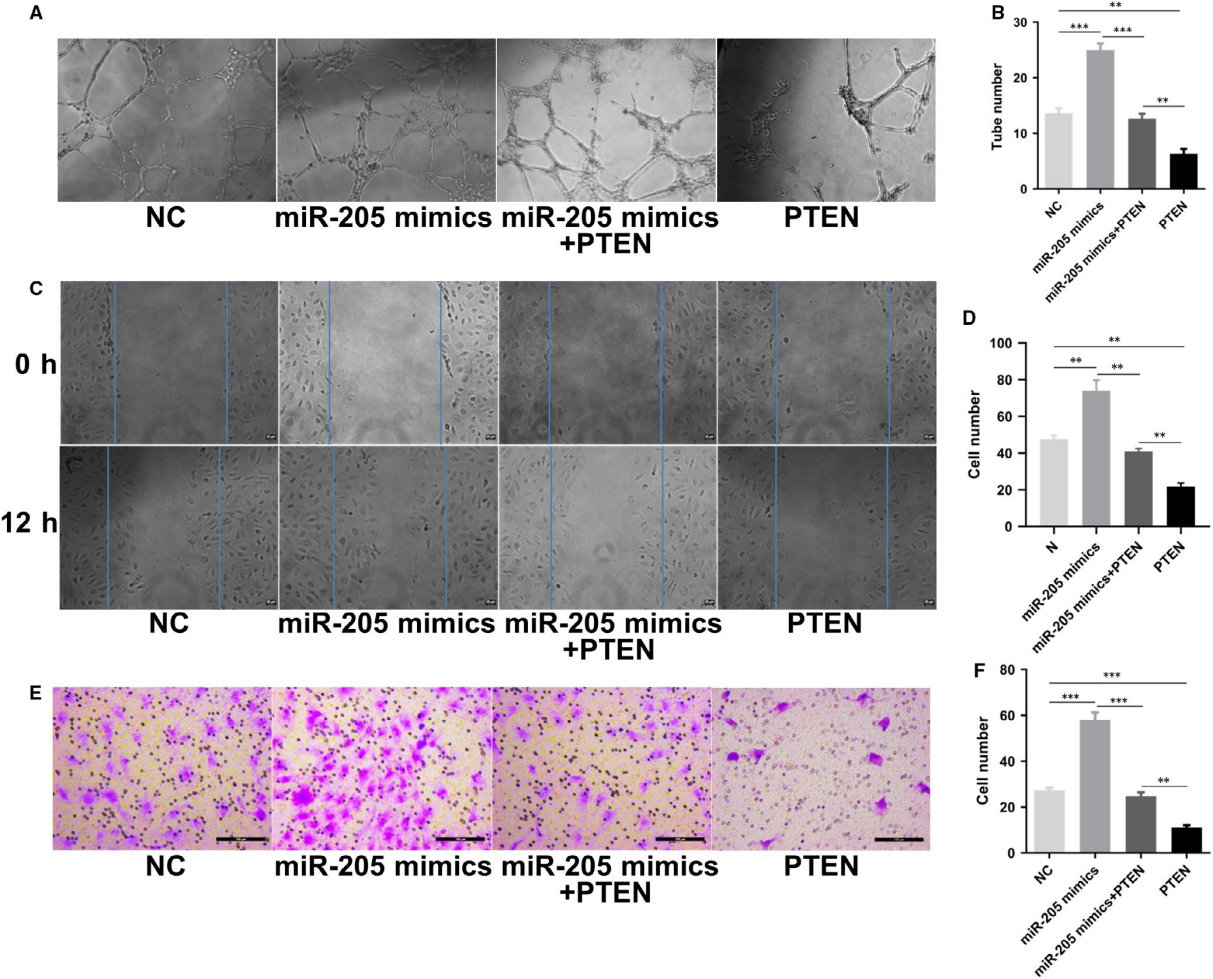
Effects of cotransfection of miR‐205 and PTEN on tube formation, cell migration and invasion of EPCs. A‐B, In vitro tube formation ability was analysed (magnification, ×100). C‐D, The migration ability of EPCs was detected by wound healing assay. Scale bar = 20 μm. E‐F, The number of invasion cells was observed by Transwell invasion assay. Scale bar = 100 μm. **P < .01, ***P < .001

To test the effects of cotransfection of miR‐205 mimics and PTEN on EPC function, tube formation, migration and invasion assay were performed. The tube formation experiment demonstrated that miR‐205 mimics markedly enhanced tube formation ability, and PTEN significantly suppressed tube formation in EPCs, compared with the NC group. Furthermore, the tube formation capacity of EPCs in the miR‐205 mimics + PTEN cotransfection group decreased and increased compared with the miR‐205 mimics group and the PTEN group, respectively. However, there was no significant difference in tube formation of EPCs between the miR‐205 mimics + PTEN cotransfection group and the NC group (*P* > .05; Figure 6A,B). The migration experiment demonstrated that the number of migration EPCs in the PTEN group was markedly decreased, whereas in the miR‐205 mimics group, it was obviously increased than in the NC group (Figure 6C,D). Analogously, the Transwell invasion assay showed similar results, that is miR‐205 overexpression significantly promoted cell invasion, while PTEN overexpression inhibited cell invasion (Figure 5E,F). The results confirmed that miR‐205 targeted PTEN to modulate EPC angiogenesis, migration and invasion.

### 3.7 Effects of cotransfection of miR‐205 and PTEN on cell autophagy and MMP2 expression in EPCs

**Figure 7:**
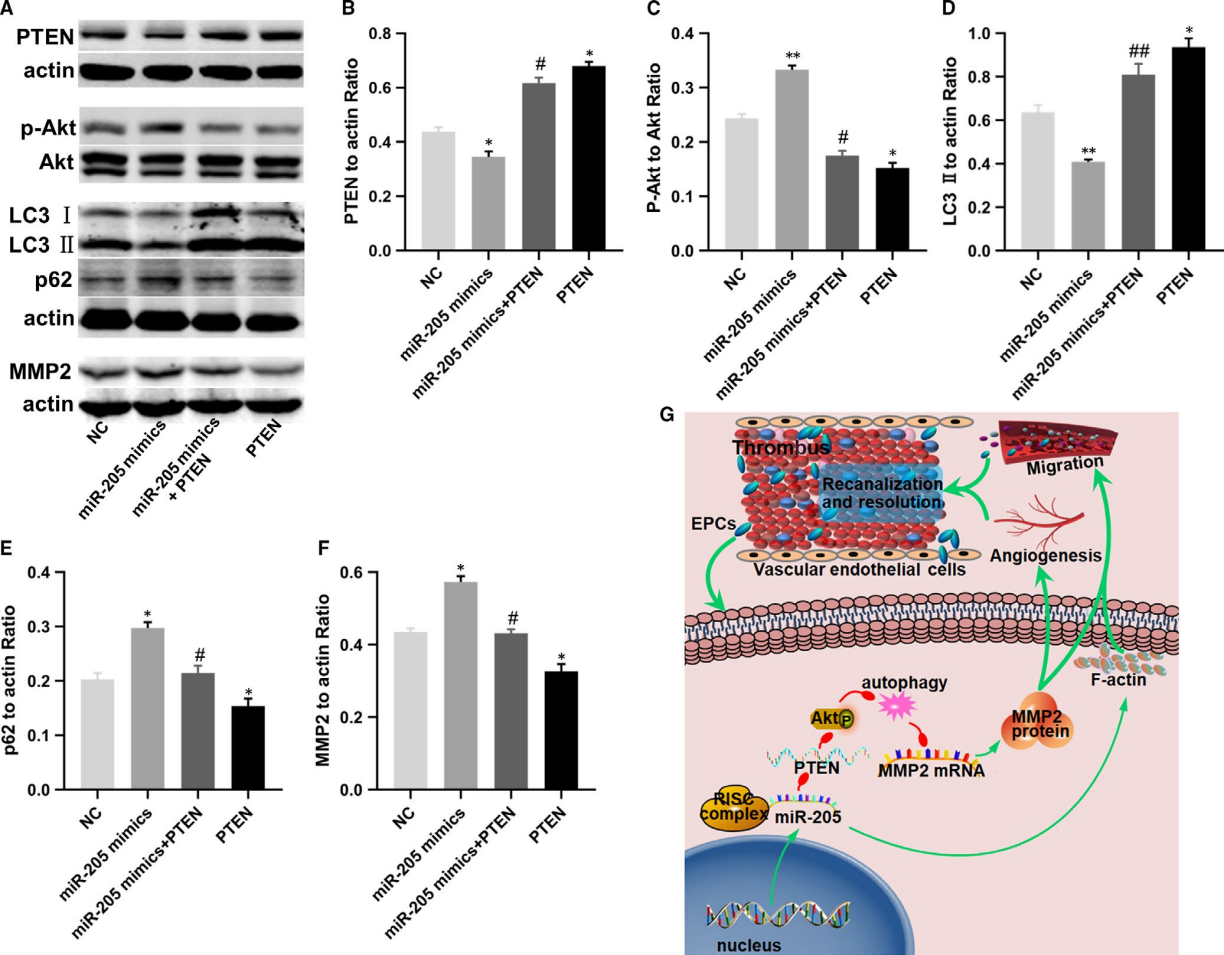
Involvement of the autophagy pathway in miR‐205‐mediated effects in EPCs on MMP‐2. A‐F, Western blot analysis of the expression of PTEN, Akt, p‐Akt, LC3‐II, p62 and MMP2 in EPCs after transfected with NC and miR‐205 mimics or cotransfected with miR‐205 mimics and PTEN, and PTEN overexpression plasmid. MiR‐205 overexpression up‐regulated the expression of Akt phosphorylation, p62 and MMP2, and inhibited PTEN and LC3 II in EPCs compared with NC group, while cotransfection of miR‐205 mimics and PTEN in EPCs could effectively antagonize miR‐205‐induced autophagy suppression and the expression of p‐Akt and MMP2, and overexpression of PTEN reduced the expression levels of Akt phosphorylation, p62 and MMP2, and increased PTEN and LC3 II in EPCs. *P < .05, **P < .01 compared with NC group; #*P* < .05, ##*P* < .01 compared with miR‐205 mimics + PTEN group. G, Principle signalling pathways of miR‐205 up‐regulation involved in promoting DVT recanalization and resolution, and EPC angiogenesis. MiR‐205 enhanced angiogenesis, migration and invasion in EPCs by directly targeting PTEN, regulating MMP‐2 mRNA and protein expression via Akt/autophagy pathway in EPCs. Additionally, F‐actin filaments were involved in the migration ability of miR‐205 in EPC

It has been shown that PTEN is a negative regulator of the PI3K/Akt pathway.^41^ The Akt pathway is closely related to cell autophagy and MMP2 expression.^21^ In addition, venous thrombus resolution and recanalization, and the migration and angiogenic behaviour of EPCs are involved in the autophagy pathway and MMP2 expression. As shown in Figures 1G and 3F‐H, MMP2 expression was up‐regulated in the thrombus sites of nude mice injected with GFP‐miR‐205 EPCs and in miR‐205‐overexpressing EPCs. As PTEN is a target of miR‐205 in EPCs, it was tempting to speculate that PTEN may mediate the regulatory effects of miR‐205 on autophagy and MMP2 expression in vitro. To test this hypothesis, EPCs were transfected with miR‐205 mimics or cotransfected with miR‐205 mimics and PTEN, and then, the protein expression of PTEN, p‐Akt, Akt, LC3I/II, p62 and MMP2 was examined by Western blot analysis (Figure 7A). As shown in Figure 7B‐F, miR‐205 overexpression inhibited PTEN expression, promoted Akt phosphorylation, suppressed autophagic flux and enhanced the expression of MMP2 in EPCs, while PTEN overexpression in EPCs had the opposite effects. Importantly, cotransfection of miR‐205 mimics and PTEN in EPCs effectively antagonized the miR‐205‐induced autophagy suppression and the expression of MMP2. The results indicated that miR‐205 regulated MMP2 via Akt/autophagy pathway by targeting PTEN.

## 4 DISCUSSION

In the present study, we identified the role of miR‐205 in DVT recanalization and resolution, and revealed its regulatory functions and molecular mechanisms in EPCs (Figure 7G). The results demonstrated that miR‐205 overexpression in EPCs significantly reduced thrombus size and weight, and facilitated venous thrombus recanalization and resolution in nude mice via enhancing EPC homing, cell growth, angiogenesis and F‐actin filaments and MMP2 expression. However, miR‐205 inhibition in EPCs exerted the opposite effects. Moreover, this study showed that PTEN was the target of miR‐205 and Akt/autophagy pathway is closely related to this process.

In recent years, multiple miRNAs have been reported to play a vital role in angiogenesis, cell growth and migration.^26^ Our previous studies have demonstrated that miRNAs have a vital role in EPC function.^11^, ^27^, ^28^, ^29^, ^30^ Additionally, miR‐205 has been shown to have different effects on angiogenesis and migration in different types of tumours.^32^, ^33^, ^34^, ^35^, ^42^ Meanwhile, miR‐205 promotes wound healing in human corneal epithelial cells.^31^ However, the role and underlying mechanism of miR‐205 in DVT and EPCs, particularly in regulating angiogenesis and thrombus resolution, remain to be elucidated.

Studies have shown that blood vessels are rich in stem/progenitor cells expressing the surface marker CD34, a marker for evaluating angiogenesis.^43^, ^44^, ^45^ Moreover, MMPs play a crucial role in degradation of the extracellular matrix and basement membrane components to allow the migration and invasion of leucocytes and other cell types in vascular diseases and homeostasis.^39^, ^46^ In particular, MMP2 is an essential factor for promoting early venous thrombus resolution.^38^, ^39^ Therefore, the expression of CD34 and MMP2 in the venous thrombus was examined by immunofluorescence. Here, we showed that miR‐205 overexpression in EPCs significantly reduced thrombus size and weight, and enhanced EPC homing and the expression of CD34 and MMP2. Therefore, we speculated that miR‐205 may promote DVT recanalization and resolution, at least in part, via enhancing angiogenesis and migration in EPCs.

To test our hypothesis, in vivo angiogenesis experiments, cell function experiments and molecular biology experiments were carried out. Our results showed that miR‐205 overexpression significantly promoted angiogenesis in vivo and in vitro, migration and invasion in EPCs. Cell migration has been shown to be strongly connected to attachment of the leading edge, cellular contraction by myosin inserting between F‐actin filament bundles and detachment of the adhesions at the trailing cell rear, which requires F‐actin for each step of the process.^47^ Additionally, MMP2 is a key regulator of EPC migration.^40^ To detect whether miR‐205 influences EPC migration by regulating F‐actin filaments and MMP2, we performed immunofluorescence of F‐actin, and qRT‐PCR and Western blot of MMP2. As shown in Figure 3E, miR‐205 inhibitor impaired F‐actin filaments, while miR‐205 mimics prevented disruption of F‐actin filaments. Analogously, we found that the expression levels of MMP2 mRNA and protein were higher upon miR‐205 overexpression in EPCs, which is consistent with the result of MMP2 immunofluorescence in thrombus sites, while miR‐205 inhibitor observably reduced MMP2 mRNA and protein levels in EPC. These findings collectively indicated that miR‐205 promoted EPC migration and invasion via regulating F‐actin filaments and MMP2, to some extent.

More importantly, in this study, dual‐luciferase reporter assays validated that PTEN is a target of miR‐205. At the same time, miR‐205 overexpression significantly inhibited the mRNA and protein expression of PTEN, while inhibition of miR‐205 clearly increased PTEN mRNA and protein expression levels. PTEN is a tumour suppressor of human cancer and has critical roles in the regulation of tumorigenesis and cell migration. Studies have shown that PTEN plays a crucial role in regulating the PI3K/Akt signalling pathway and that activation of Akt inhibits the autophagy pathway.^48^, ^49^ Furthermore, PTEN has been reported to play a major role in inhibiting migration and invasion of tumour cell via the Akt/SP1/MMP2 pathway.^50^ Intriguingly, autophagy inhibition promoted EPC migration by up‐regulating MMP2 expression.^21^, ^40^ In addition, it has been verified that miR‑205 enhanced ovarian cancer cell invasion by regulating MMP2/9 expression in vitro.^37^ Thus, we examined the effects of miR‐205 overexpression, co‐expression of miR‐205 and PTEN, and PTEN overexpression on PTEN/Akt/autophagy pathway and MMP2 expression in EPCs. Our results showed that co‐expression of miR‐205 and PTEN in EPCs effectively antagonized the miR‐205‐induced autophagy suppression, MMP2 expression and EPC functions. Thus, this study revealed that miR‐205 targeted PTEN, to regulate MMP2 expression via Akt/autophagy pathway, thereby mediating angiogenesis, cell growth and migration of EPCs, subsequently enhancing the ability of EPC to homing to the thrombus to accelerate venous thrombus recanalization and resolution.

In summary, we demonstrate that miR‐205 directly targets PTEN to modulate MMP2 expression via the Akt/autophagy pathway, thus playing a key role in EPC function and DVT recanalization and resolution. This study provides valuable information for exploring miRNAs in the treatment of DVT.

## CONFLICT OF INTEREST

The authors confirm that there is no conflict of interests.

## AUTHOR CONTRIBUTIONS

Li‐Li Sun: designed the research and drafted the manuscript. Lun Xiao, Xiao‐Long Du and Lei Hong: performed the experiments and analysed the data. Cheng‐Long Li and Jian Jiao: assisted with research design and data analysis. Wen‐Dong Li and Xiao‐Qiang Li: performed the research and critically revised the manuscript. All authors have reviewed and approved the final manuscript.

## Data Availability

The data used to support the findings of this study are available upon reasonable request from the corresponding authors.
